# Unnatural Deaths in South African Platinum Miners, 1992–2008

**DOI:** 10.1371/journal.pone.0022807

**Published:** 2011-09-13

**Authors:** Megan S. C. Lim, Jill Murray, Robert J. Dowdeswell, Judith R. Glynn, Pam Sonnenberg

**Affiliations:** 1 Research Department of Infection and Population Health, University College London, London, United Kingdom; 2 National Institute for Occupational Health, National Health Laboratory Service, Johannesburg, South Africa; 3 School of Public Health, University of the Witwatersrand, Johannesburg, South Africa; 4 Rustenburg Platinum Mines Limited: Precious Metals Refiners, Rustenburg, South Africa; 5 London School of Hygiene and Tropical Medicine, London, United Kingdom; Finnish Institute of Occupational Health, Finland

## Abstract

**Background:**

The mortality rate from unnatural deaths for South Africa is nearly double the world average. Reliable data are limited by inaccurate and incomplete ascertainment of specific causes of unnatural death. This study describes trends in causes of unnatural death between 1992 and 2008 in a cohort of South African miners.

**Methodology/Principal Findings:**

The study used routinely-collected retrospective data with cause of death determined from multiple sources including the mine's human resources database, medical records, death registration, and autopsy. Cause-specific mortality rates and Poisson regression coefficients were calculated by calendar year and age group. The cohort included 40,043 men. One quarter of all 2937 deaths were from unnatural causes (n = 805). Causes of unnatural deaths were road traffic accidents 38% (109/100,000 py), homicides 30% (88/100,000 py), occupational injuries 17% (50/100,000 py), suicides 8% (24/100,000 py), and other accidents 6% (19/100,000 py). Rates of unnatural deaths declined by 2% (95%CI -4%,-1%) per year over the study period, driven by declining rates of road traffic and other accidents. The rate of occupational injury mortality did not change significantly over time (-2% per year, 95%CI -5%,+2%). Unnatural deaths were less frequent in this cohort of workers than in the South African population (IRR 0.89, 95%CI 0.82–0.95), particularly homicides (IRR 0.48, 95%CI 0.42–0.55).

**Conclusions/Significance:**

Unnatural deaths were a common cause of preventable and premature death in this cohort of miners. While unnatural death rates declined between 1992 and 2008, occupational fatalities remained at a high level. Evidence-based prevention strategies to address these avoidable deaths are urgently needed.

## Introduction

Deaths from unnatural causes are largely preventable and generally occur in young people resulting in a substantial loss of potential life. The mortality rate of unnatural deaths was estimated by WHO in 2004 to be 90/100,000 worldwide and 104/100,000 in the African region.[1] Rates are particularly high in South Africa, where the WHO report mortality rates from unnatural causes of nearly double the world average (159/100,000).[2] Unnatural causes are the second leading cause of death in South Africa after HIV/AIDS.[3] Extremely high levels of interpersonal violence, traffic injuries and other accidents are major drivers of this trend.[4], [5] Men are particularly affected by unnatural deaths with a higher proportion of deaths among young adults due to unnatural causes (21% for males and 5% for females) and eight times as many homicides among men than among women.[6] South African men of working age suffer from the second highest rate of homicide and the 12^th^ highest rate of road traffic accident mortality of men in any country in the world.[1]

Although routine statistics provide a broad overview, accurate data on specific causes of unnatural death in South Africa are limited. For example, vital registration assigned ‘events of undetermined intent’ as the cause of two-thirds of all reported unnatural deaths in 2006.[6] The National Injury Mortality Surveillance System reports more detailed data on a selection of unnatural deaths, but is not representative of all deaths, and in the most recent report (2008), 9% of unnatural deaths were of undetermined causality.[7] Currently the death notification form does not make provision for the manner of death from injuries e.g. homicide, suicide or accident.[8] Detailed estimates of the burden of disease in South Africa were made in a study in 2000, but changes in unnatural death rates over time have not been determined.[9]

While unnatural deaths are still an unacceptably common cause of death in South Africa, there has been some suggestion that this has decreased over time.[6],[10] In South Africa over the last few decades, deaths due to natural causes have consistently increased over time, due to rapidly rising mortality from HIV/AIDS. With the HIV/AIDS epidemic presenting an increasingly major competing cause of death, the proportion of deaths due to unnatural causes has declined (from 17% in 1997 to 9% in 2008).[6] Data on mortality rates, however, have shown a steady rate of unnatural deaths over time.[11]

This study aims to describe the rate of unnatural deaths, the causes of death and trends over time in a cohort of men employed in a South African platinum mine between 1992 and 2008. These miners face the risk of death from occupational injuries, and are also subject to the risks of other violent and accidental deaths facing all South African men. The availability of comprehensive data on deaths, including clinical and autopsy cause of death on a large proportion of working men, provides a rare opportunity to investigate unnatural deaths occurring both within and outside the workplace. The findings of this study will add information to existing routine statistics, to improve our picture of the causes and trends in unnatural deaths during the past two decades in South Africa. Accurate statistics are the first step in developing targeted interventions to prevent unnatural deaths.

## Materials and Methods

The setting for the study was a large platinum mine in North West Province, South Africa, employing approximately 22,000 people in 1992.

The study used routinely-collected retrospective data; individuals were linked by a unique company number. The data sources included a personnel human resources database with details of employment dates; the mine hospital death register; provident fund records; and the National Institute for Occupational Health autopsy database. All deaths occurring during employment are reported in personnel records, maintained by the company. The most likely underlying cause of death was determined using all available data sources. Autopsies performed prior to 1996 were not available. Autopsies, conducted for compensation purposes with the written consent of next of kin, were of heart and lung only and were conducted regardless of the clinical cause of death. Overall, 46% of all deaths and 49% of unnatural deaths had an autopsy conducted; the proportion of deaths with an autopsy was 70% for occupational injuries, 32% for road traffic accidents, 56% for homicide, 47% for suicide, and 53% for other accidents.

Mine personnel records were used to construct an open cohort of all semiskilled and unskilled male miners who worked at the mine between 1992 and 2008. Skilled workers were excluded as they receive medical care elsewhere and thus their data would be incomplete. Subjects entered the cohort on 1 January 1992 or on the date of employment if later than this. They remained in the cohort until they died, left the mine or until 31 December 2008.

Data were entered onto computer, cleaned and all patient-identifying variables removed. Individual records were expanded into person years (py) at risk by calendar year (1992 to 2008) and age categories. Cause-specific mortality rates were calculated by calendar year and age group. Direct age standardisation was applied using 10 year age groups, and taking the age distribution of the Black African male adult (20 to 69 years) South African population [12] as the standard. Age-adjusted Poisson regression coefficients determined the average change in rate per year during the study period since 1992. Age-specific and cause-specific mortality rates were compared to rates among all adult males in the South African population in 2000, as reported by Bradshaw et.al.[9] All analyses were conducted in Stata 10.

Ethical approval was obtained from ethics committees of the University of the Witwatersrand, South Africa, and University College London, United Kingdom.

## Results

The cohort consisted of 40,043 men and 278,993 person years (py). The median length of time spent employed at the mine was 10.2 years. 98% were black African and the median age at entry was 35.1 years. There were 2937 deaths in the cohort overall; a mortality rate of 1051/100,000 py.

There were 808 (27%) deaths from unnatural causes between 1992 and 2008, a rate of 290/100,000 py (95%CI 270–310). This was the second most common cause of death after HIV/AIDS (44%, 466/100,00 py). Among men who died who were HIV negative or of unknown status (n = 1539), half (n = 750) the deaths were due to unnatural causes. Rates of unnatural deaths by HIV status could not be calculated as the HIV status of living individuals is not known.

Road traffic accidents were the most common cause of unnatural death (38%), followed by homicides (30%) and occupational injuries (17%) (Table 1).

**Table 1:** Cause of unnatural death.

Year	All Unnatural causes			Occupational injury			Road traffic accident			Other accident			Homicide			Suicide		
	n	Unadjusted rate	Standardised rate	n	Unadj rate	Stand rate	n	Unadj rate	Stand rate	n	Unadj rate	Stand rate	n	Unadj rate	Stand rate	n	Unadj rate	Stand rate
1992	48	249.7	223.7	10	52.0	32.0	26	135.3	102.2	0	0.0	0.0	9	46.8	35.6	2	10.4	49.1
1993	59	311.7	299.1	13	68.7	48.2	20	105.6	81.1	5	26.4	21.0	18	95.1	141.5	2	10.6	4.8
1994	66	345.1	418.6	12	62.7	51.3	33	172.5	303.3	7	36.6	23.2	11	57.5	34.0	4	20.9	10.8
1995	58	301.8	266.4	11	57.2	41.1	22	114.5	90.4	5	26.0	14.0	16	83.3	107.2	5	26.0	17.4
1996	55	294.5	334.8	6	32.1	14.7	21	112.4	107.2	6	32.1	19.3	17	91.0	167.7	5	26.8	25.9
1997	63	342.0	295.7	11	59.7	67.4	24	130.3	105.0	9	48.9	56.4	13	70.6	52.2	5	27.1	12.5
1998	60	338.9	310.9	7	39.5	35.7	21	118.6	81.7	1	5.6	3.3	28	158.1	183.3	3	16.9	7.0
1999	48	280.7	368.1	4	23.4	10.9	19	111.1	123.9	2	11.7	14.8	18	105.3	186.3	5	29.2	32.1
2000	68	413.5	388.6	8	48.6	63.2	23	139.9	62.0	6	36.5	37.2	23	139.9	172.5	8	48.6	53.6
2001	57	370.2	460.5	6	39.0	125.3	27	175.4	102.4	2	13.0	5.7	15	97.4	168.1	7	45.5	59.1
2002	48	317.7	327.8	12	79.4	69.5	9	59.6	87.1	1	6.6	3.3	21	139.0	84.7	5	33.1	83.2
2003	32	208.9	189.4	7	45.7	25.3	7	45.7	17.8	3	19.6	14.6	11	71.8	76.6	3	19.6	45.1
2004	32	216.1	165.1	5	33.8	15.1	6	40.5	30.6	2	13.5	8.4	14	94.5	92.1	5	33.8	19.0
2005	23	186.4	144.0	5	40.5	30.9	10	81.0	53.7	1	8.1	5.3	7	56.7	54.1	0	0.0	0.0
2006	21	180.7	159.7	4	34.4	43.6	11	94.6	69.2	0	0.0	0.0	5	43.0	35.0	1	8.6	12.0
2007	40	286.3	348.0	14	100.2	93.7	14	100.2	102.3	0	0.0	0.0	7	50.1	111.6	5	35.8	40.4
2008	30	192.6	147.0	3	19.3	19.8	11	70.6	56.5	2	12.8	11.2	11	70.6	43.8	3	19.3	15.6
**TOTAL**	**808**	**289.6**	**279.6**	**138**	**49.5**	**43.5**	**304**	**109.0**	**96.9**	**52**	**18.6**	**15.0**	**244**	**87.5**	**97.9**	**68**	**24.4**	**25.3**
Trend*	-2.3% per year (-3.7%,-0.8%)			-2.2% per year (-5.7%,+1.4%)			-4.6% per year (-7.0%,-2.1%)			-7.8% per year (-14.0%,-1.7%)			+0.1% (-2.5%,+2.7%)			+2.4% (-2.4%,+7.3%)		

Table 2 shows cause-specific rates of mortality by year. Rates of unnatural deaths declined by an average of 2% (95%CI -4%,-1%) per year over the study period. Road traffic accident and other accident mortality rates declined significantly over time. The incidence of occupational injury fatalities, homicides, and suicides in the population did not change significantly over time.

**Table 2:** Number of deaths and cause-specific mortality rates per 100,000 py, by calendar year.

Cause of death	Age group	deaths (n)	Rate per 100,000 py	Rate ratio (95%CI) by age group	South African male population rate (2000) [28]	Rate ratio (95%CI) Cohort vs South African population
**All unnatural**	**All ages**	**808**	**289.6**		**327.1**	**0.89 (0.82–0.95)**
	15–24	19	279.1	1.00	273.4	1.02 (0.61–1.60)
	25–34	160	265.9	0.91 (0.57–1.47)	405.9	0.66 (0.56–0.77)
	35–44	352	301.5	1.05 (0.66–1.66)	392.2	0.77 (0.69–0.85)
	45–54	237	299.2	1.10 (0.69–1.75)	291.6	1.03 (0.90–1.17)
	55+	36	224.2	0.82 (0.47–1.42)	227.3	0.99 (0.69–1.37)
**Homicide**	**All ages**	**244**	**87.5**		**182.0**	**0.48 (0.42–0.55)**
	15–24	10	146.9	1.00	178.8	0.82 (0.39–1.51)
	25–34	54	89.8	0.61 (0.31–1.20)	246.8	0.36 (0.27–0.48)
	35–44	105	89.9	0.61 (0.32–1.17)	206.7	0.44 (0.36–0.53)
	45–54	68	85.8	0.58 (0.30–1.13)	121.4	0.71 (0.55–0.90)
	55+	5	31.1	0.21 (0.07–0.62)	83.6	0.37 (0.12–0.87)
**Suicide**	**All ages**	**68**	**24.4**		**32.4**	**0.75 (0.58–0.96)**
	15–24	2	29.4	1.00	25.4	1.16 (0.14–4.19)
	25–34	17	28.3	0.96 (0.22–4.16)	37.4	0.75 (0.44–1.21)
	35–44	32	27.4	0.93 (0.22–3.89)	34.1	0.80 (0.55–1.14)
	45–54	15	18.9	0.64 (0.15–2.82)	37.5	0.51 (0.28–0.84)
	55+	2	12.4	0.42 (0.06–3.01)	28.7	0.43 (0.05–1.58)
**Occupational injury**	**All ages**	**138**	**49.5**		**2.2**	**22.6 (18.4–27.7)**
	15–24	2	29.4	1.00	0.6	50.5 (5.79–202.3)
	25–34	22	36.6	1.24 (0.29–5.29)	1.4	25.1 (14.6–41.9)
	35–44	59	50.5	1.72 (0.42–7.04)	5.8	8.72 (6.36–11.79)
	45–54	47	59.3	2.02 (0.49–8.31)	3.6	16.4 (11.1–24.1)
	55+	10	62.3	2.12 (0.46–9.67)	0.0	––
**Road traffic accident**	**All ages**	**304**	**109.0**		**76.8**	**1.42 (1.26–1.59)**
	15–24	5	73.5	1.00	48.4	1.52 (0.49–3.55)
	25–34	58	96.4	1.31 (0.53–3.27)	87.2	1.11 (0.84–1.43)
	35–44	135	115.6	1.57 (0.64–3.84)	103.5	1.12 (0.93–1.33)
	45–54	96	121.2	1.65 (0.67–4.05)	86.5	1.40 (1.13–1.72)
	55+	14	87.2	1.19 (0.43–3.29)	73.8	1.18 (0.64–1.99)
**Other accident**	**All ages**	**52**	**18.6**		**33.7**	**0.55 (0.41–0.73)**
	15–24	0	0.0	–––	45.6	0.0 (0.0–2.69)
	25–34	9	15.0	1.00	70.5	0.45 (0.21–0.86)
	35–44	19	16.3	1.09 (0.49–2.40)	76.2	0.38 (0.23–0.60)
	45–54	10	12.6	0.84 (0.34–2.08)	80.1	0.70 (0.45–0.86)
	55+	4	24.9	1.66 (0.51–5.41)	69.9	0.60 (0.16–1.56)

Rates are standardised to the population distribution of adult men in the South African population [12]

* refers to average change per year in rate of cause-specific death.

Cause-specific rates of unnatural death by age group are shown in Table 3. The rates of death due to occupational injuries showed a non-significant increase with age. Rates of homicide were highest in men under 25 years, at 147/100,000. The rate of homicide and suicide decreased with increasing age.

**Table 3:** Cause-specific mortality rates, by age group, and comparison to South African male population mortality rate in adult men aged over 15 years.

Cause of death		n	%	rate per 100 000 py (95%CI)
**Occupational**	**Occupational injury**	**138**	**17.1**	**49.5 (41.7-58.4)**
**Accidental**	**TOTAL accidents**	**356**	**44.0**	**127.6 (114.7-141.6)**
	Road traffic accident	304	37.6	109.0 (97.1-121.9)
	Accidental poisoning	20	2.5	7.2 (4.4-11.1)
	Fire	12	1.5	4.3 (2.2-7.5)
	Drowning	8	1.0	2.9 (1.2-5.7)
	Pedestrian struck by train	7	0.9	2.5 (1.0-5.2)
	Lightning	1	0.1	0.4 (0.0-2.0)
	Fall	1	0.1	0.4 (0.0-2.0)
	Complications of medical care	2	0.2	0.7 (0.1-2.6)
**Homicide**	**TOTAL homicides**	**244**	**30.2**	**87.5 (76.8-99.2)**
	Firearm	129	15.9	46.2 (38.6-54.9)
	Sharp object/stabbing	37	4.6	13.3 (9.3-18.3)
	Other/unspecified	78	9.6	28.0 (22.1-34.9)
**Suicide**	**TOTAL suicides**	**68**	**8.4**	**24.4 (18.9-30.9)**
	Hanging	52	6.4	18.6 (13.9-24.4)
	Firearm or explosive	6	0.7	2.1 (0.8-4.7)
	Other/unspecified	10	1.2	3.6 (1.7-6.6)
**Other/Unspecified**		3	0.4	1.1 (0.2-3.1)
**TOTAL**		**808**	**100**	289.6 (270.0-310.3)

Age and cause specific mortality rates are compared to estimated rates in the South African adult male population in 2000 (Table 3). Men in this cohort had lower rates of unnatural deaths overall than the average South African population (IRR 0.89, 95%CI 0.82–0.95). Road traffic accidents and occupational fatalities were more common in this cohort than in population estimates, whereas other causes of unnatural deaths were less frequent.

## Discussion

Unnatural deaths were a very common cause of death in this cohort of South African platinum miners, resulting in 808 premature deaths in a 17-year period, one quarter of all deaths in the cohort. The overall rate of unnatural death in this cohort was 290/100,000 py, more than triple the worldwide average for adult males in 2004 (90/100,000 py).[1] Comparison to age matched mortality rates estimated for South Africa in 2000 show that men in this cohort had lower rates of unnatural deaths overall than the average South African population (IRR 0.89, 95%CI 0.82–0.95).[9] This was largely driven by lower rates of homicide, suicides, and accidents in the cohort of miners. Significantly higher rates of occupational fatalities and road traffic accidents went some way to diluting this overall effect.

An occupational cohort is not be representative of the entire population, as the “healthy worker effect”, the hazards of work in the mining industry and migration for work may all have affected rates of mortality. Nevertheless, this is an important group to study as the premature deaths of these men and the loss of their income potential have broad implications for their families and communities; it is estimated that each miner supports between 7 and 10 dependents.[13] The study excluded women (because numbers were small), who face different issues such as intimate partner violence and sexual violence.[5], [6]

Strengths of this study include the long time period covered (17 years) and the large size of the cohort, which was not confined to those attending a health service or consenting to participate in a research study, minimising selection bias. Data completeness was good and accuracy of ascertainment of causes of death was very high because linkage has been possible across a wide range of sources. We were able to attribute a specific cause of unnatural death to 99.5% of the deaths. Ascertainment of the rate of mining fatalities is particularly robust due to South African Department of Mineral Resources investigations into all serious mining injuries and fatalities. Furthermore, this study was unique in being able to calculate mortality rates using an accurate denominator, which is not often possible in other surveillance systems.[6], [7]

There was a high rate of occupational fatalities in our cohort over the entire study period (49.5/100,000 py), and although there was a decrease of 2.2% per year, this was not statistically significant. Data from the South African Department of Mineral Resources show a significant decline in platinum mine occupational fatalities (from 49/100,000 workers in 1992 to 19/100,000 workers in 2008), gold mine occupational fatalities (from 113/100,000 workers in 1992 to 55/100,000 workers in 2008), and occupational fatalities in the South African mining industry as a whole (from 94/100,000 workers in 1992 to 35/100,000 workers in 2008) (Personal communication, M. Hugo, Mine Health and Safety Inspectorate, South African Department of Mineral Resources). Studies from other African mines have reported even higher mortality rates from occupational injuries than in this cohort, such as 110/100,000 py in South African HIV-negative gold miners between 1991 and 2002,[14] 111/100,000 py in a Zambian copper mine in 2005–07,[15] and 83/100,000 py in Zimbabwean mines in 1995, much higher rates than in the mining industry in industrialised countries.[16] For example, in the USA the rate of occupational fatalities in the mining industry was 12.4/100,000 workers in 2009, one quarter of the rate in this cohort.[17] However, there are significant limitations in comparing fatality rates from different mining settings, as different levels of depth and proportion of workers underground alter risk rates.[18] By comparison, the reported occupational fatality rate in South Africa overall was much lower, at 14/100,000 workers in 1993.[16]

In 2003, an analysis of fatal occupational injuries in the platinum mining sector in South Africa, that included the cohort reported here, showed that 70% of deaths were related to compliance issues.[19] In response, the industry implemented behaviour-based safety programmes in addition to the safety management systems traditionally used in the industry. Still, despite improving technology and greater industry emphasis on safety, occupational mining deaths are unacceptably frequent.

Occupational injury mortality rates increased with age, in contrast to a previous study in South African gold miners which found a decrease in work-related injuries in those aged over 50 years.[14] This study of gold miners also showed that HIV positivity was associated with an increase in work-related injury rates (but not fatalities).[14] HIV/AIDS was the most common cause of death in this cohort, as with other mining cohorts [20] and in South Africa as a whole.[9]

The rate of road accidents deaths in this cohort was significantly higher than among men of the same age in the South African population in 2000 (IRR 1.42, 95% CI 1.26–1.59). However, the rate of road traffic accident deaths decreased by an average of 4% per year over the study period, from very high rates of over 100/100,000 py for each year until 2001. One possible explanation for the decrease in these deaths is that over time employees (and their families) have become more likely to live closer to the mine, reducing their need to travel home on weekends and holidays using dangerous buses and highways. Rates of personal car ownership have also increased, possibly resulting in increased care-taking on the roads. However, these accidents still remain the most common cause of unnatural death in this cohort. Alcohol misuse, excessive speeding, driver fatigue, mechanical failures and inadequate facilities for pedestrians are reported to play a large role in traffic deaths in South Africa.[5], [21]–[23] South Africa's road traffic accident mortality rate is estimated to be 26% higher than the average for the African region and nearly double the global rate.[5]

South Africa has one of the highest homicide rates in the world [24] but in this cohort the homicide rate was half that in the general population (IRR 0.48, 95%CI 0.42–0.55). Violence in South Africa is largely a manifestation of broad social and structural factors and is driven by poverty, unemployment, inequity, racial and political conflict, social dynamics, lack of firearm control, and high levels of drug and alcohol use.[5], [24], [25] Temporal trends in homicides in our study did not match linear decreases (from 67/100,000 in 1994/5 to 43/100,000 in 2003/4 and 34/100,000 in 2009/10) reported by the South African Police Service.[26], [27] Deaths from homicide were particularly prevalent in young men aged less than 25 years, with 147 homicide deaths per 100,000 py. Firearms were the most common mode of homicide. Suicides were also more prevalent in the youngest age group, relative to older age groups, and were also less common in this cohort than the general population (IRR 0.75, 95%CI 0.58–0.96). The impact of employment on violence in South Africa warrants further elucidation.

Unnatural deaths were a very frequent cause of preventable death from 1992 to 2008 in this cohort of over 40,000 miners. These miners are working men whose premature deaths will have broad personal and economic impacts on their families and communities. The majority of unnatural deaths were the result of road traffic accidents or homicide. The rate of occupational fatalities in this cohort was much higher than among South African workers as a whole, and there has been no significant decline in these deaths over time despite improving technology and greater industry emphasis on safety. A comprehensive, evidence-based injury prevention strategy that includes industry, workers, government and communities is urgently needed to address injury deaths occurring inside the workplace and out. Effective strategies such as use of seatbelts, drug and alcohol harm reduction, and gun control need support at a local level. Further evaluation of the implementation of different preventative strategies, including prevention trials and analysis of larger employee cohorts or non-fatal injuries, is warranted to determine which approaches are most effective. Providing these accurate data, allowing triangulation with public statistics, is one step in creating targeted interventions to address this important problem.
